# Predictive factors of vaccination status, knowledge, attitudes, and practice towards prevention of hepatitis B infection among Bangladeshi people: A cross‐sectional study

**DOI:** 10.1002/hsr2.1000

**Published:** 2022-12-19

**Authors:** Ismail Hosen, Mst. Sabrina Moonajilin, Nur Hussain

**Affiliations:** ^1^ Department of Public Health and Informatics Jahangirnagar University Savar Dhaka Bangladesh; ^2^ Department of Epidemiology CHINTA Research Bangladesh Savar Dhaka Bangladesh; ^3^ School of Earth, Environment & Society McMaster University Hamilton Ontario Canada

**Keywords:** attitude, Bangladesh, hepatitis B, knowledge, practice, vaccination status

## Abstract

**Background and Aims:**

Infection with the hepatitis B virus is a serious public health problem that is growing all over the world. Therefore, in this context, there is no exception to public participation in disease burden reduction. Consequently, for the first time in Bangladesh, the current study aims to assess the level of vaccination status, knowledge, attitude, and practice of hepatitis B infection among general people.

**Methods:**

A cross‐sectional study was carried out between December 15, 2021, and January 17, 2022, including sociodemographic information as well as questions about vaccination status and knowledge, attitude, and practice related to hepatitis B. Data were analyzed using descriptive (frequency) and inferential statistics (Mann–Whitney *U*, Kruskal–Wallis *H*, *χ* ^2^, binary logistic regression, and spearman's rho correlation coefficient).

**Results:**

Results indicated that about one‐third (37.9%) of the 807 participants had received hepatitis B vaccine, with an overall mean score of 11.506 ± 5.403 for knowledge, 5.435 ± 1.038 and 4.252 ± 1.776 for attitude and practice, respectively. Risk factors related to vaccination were age, religion, educational qualification, occupation, residence area, marital status, comorbidity, and family member suffering from hepatitis B. Higher level of knowledge was significantly found among the young people aged between 10 and 29; had higher secondary or tertiary education (median = 13); were employed (median = 13.5, interquartile range [IQR] = 8); living in divisional city (median = 13, IQR = 7); were single (media = 13, IQR = 7); and whose family members were suffering from hepatitis B. Besides, poor practice was observed among those aged between 50 and higher (*p* = 0.004), had no formal education [*p* < 0.001), a retired or housewife (*p* < 0.001), divorced or widowed (*p* < 0.001), absence of comorbidity (*p* = 0.02), and whose family members were not infected with hepatitis B (*p* < 0.001).

**Conclusions:**

The results exposed that vaccination rates and preventative behavior are unsatisfactory, which will hinder efforts to eradicate hepatitis B worldwide by the year 2030.

## 1 INTRODUCTION

In the healthcare setting, hepatitis B (HB) is widely considered as a global public health threat since HB is a potentially fatal viral infection caused by the hepatitis B virus (HBV).^1^, ^2^ This virus can easily be transmitted from one infected person to another, with the most common transmission routes being perinatal transmission (mother to child at birth) or horizontal transmission (exposure to infected blood).^2^, ^3^ In addition, unprotected sexual contact, sharing of eating utensils and other barber shop and beauty salon equipment, tattooing, ear piercing, acupuncture, dialysis, and even syringe use can all be potential sources of infection.^1^, ^2^, ^4^, ^5^ People infected with HB generally exhibit the common symptoms of yellowing of the skin and eyes (jaundice), dark urine, extreme fatigue, nausea, vomiting, and abdominal pain.^2^ Based on the severity, HB infection can be acute or chronic, with infants and children being more vulnerable than adults.^2^ Although acute HB can be cured with prompt medical attention, people with chronic HB such as liver disease, liver cirrhosis, and hepatic‐cellular carcinoma may experience lifelong complications and even death in extreme cases.^1^, ^6^, ^7^, ^8^

According to the World Health Organization (WHO) report 2019, approximately 296 million people have lifelong chronic HB infection and there is an ongoing increase in the detection of new cases around the world.^2^, ^9^ WHO also estimated that about 1.5 million new infections occur each year all over the world along with 820,000 deaths caused by chronic HB, primarily liver cancer.^2^ Despite the fact that HB is classified as a "priority disease” in a global context. In line with the situation, the WHO Western Pacific Region has a massive burden of HB infection, while the WHO South‐East Asia Region has 18 million.^2^ As part of South Asia, Bangladesh was reported to be one of the top 10 burdened countries for viral hepatitis due to the lack of health education, illiteracy, poverty, and a lack of HB vaccination.^10^, ^11^, ^12^ Consequently, Bangladesh has an intermediate chronic HB prevalence estimated at 2%–6% although epidemiology may vary between geographic regions and sociodemographic factors.^10^ As mentioned earlier, the prevalence of HB is increasing over the world, therefore, prevention is seen as one of the best ways to protect people's health.^9^, ^13^ There is no exception to public participation in reducing disease burden in this context, and evidence suggests that the public's health‐related behavior is determined by their knowledge, attitude, and practice (KAP).^3^ KAP studies are widely regarded as an important component of public health because they represent a specific population's health‐seeking behaviors based on what the participants know, believe, and practice about a specific disease.^3^, ^9^ Although HB is a significant occupational hazard especially for health workers,^14^ it can also be a threat for all people because of their unawareness. To reduce the HB infection among the people of Bangladesh, it is critical to understand their vaccination status, knowledge, attitude, and preventive behavior, as Bangladesh is unable to control it without public participation due to limited healthcare resources. Previously, few studies were conducted in the country in response to the issues,^5^, ^15^, ^16^, ^17^, ^18^, ^19^, ^20^ but to the best of knowledge, none of the studies evaluated the level of KAP along with its predictors among general people in Bangladesh. Therefore, the current study aims to investigate this knowledge gap. The study's main strength is that it provides a better understanding of the scenario of HB infection in Bangladesh, which is important for HBV prevention. Hence, it is anticipated that the present study findings would be helpful for the government of Bangladesh to take necessary steps based on the study findings to prevent HB infection in Bangladesh.

## 2 METHODS

### 2.1 Study procedure, participants, and ethics

Data were collected using an online‐based data collection tool (e.g., Google form) due to the outbreak of the omicron variant of the COVID‐19 pandemic in Bangladesh. Data collection was completed between December 15, 2021, and January 17, 2022. Initially, a structured questionnaire was developed and pretested among the research assistants although minor modification was done after the pilot testing. This questionnaire was distributed among the target population through utilizing diverse social media sites such as Facebook, Messenger, WhatsApp, and so on. Besides, data from illiterate people were collected through an interview. To take part in the survey, participants had to be active Bangladeshi people at least 13 years old and be interested in the study. In addition, the respondents gave their informed consent to participate in the study after learning about the study's goals and objectives (consent was provided by the legal guardians whose age was under 18). Furthermore, the data's confidentiality and anonymity were also guaranteed. Primarily, 863 respondents completed the survey where 807 data were utilized for final analysis in the study after the incomplete survey was eliminated. For all variables in our survey, "mandatory" fields were used to exclude missing data. All procedures were carried out in accordance with the Helsinki Declaration of 2013 and its subsequent amendments.^21^ Besides, the ethical aspects were also granted by the Biosafety, Biosecurity, and Ethical Committee of Jahangirnagar University, Bangladesh.

### 2.2 Sample size estimation

$$n=\frac{Z^{2}p(1-p)}{d^{2}}.$$

Here, *n* = sample size; *Z* = 1.96 at 95% confidence interval (CI); *p* = prevalence rate estimation (50% or 0.5); 1 − *p* = (1 − 0.5) = 0.5; *d* = margin of error (considered as 5% or 0.05). Through using the formula, the estimated sample size was 385.

### 2.3 Measures

The survey questionnaire included sociodemographic information, vaccination status, and knowledge, attitude, and practice toward HB infection. The detailed questionnaire was available in the attached Supporting Information file.

#### 2.3.1 Sociodemographic measure

Sociodemographic information was collected on the basis of age, gender, educational qualification, occupation, current division of residence, current living area, marital status, smoking, and alcohol consuming status, and health‐related comorbidities (e.g., diabetics, high blood pressure, asthma/respiratory problem, heart disease, kidney problem, cancer, others). In addition, participants were also asked to respond dichotomously about whether anyone in their family had HB.

#### 2.3.2 Vaccination status and knowledge, attitude, and practice

HBV vaccination status was determined by asking a single question about whether or not they had received the vaccine. The approach used in this present study to assess the level of knowledge, attitude, and practice was previously utilized in a few studies.^9^, ^18^ The approach consisted of a 35 items questionnaire where 20 questions evaluate the knowledge level, 7 items, and 8 items for attitude and practice, respectively, toward HB.^9^ Participant's responses were recorded dichotomously where knowledge, attitude, and practice were assessed by giving 1 to each correct answer and 0 to the wrong answer.

### 2.4 Data analysis

Primarily, the collected data through Google forms were cleaned and prepared by Microsoft Excel 2019 for final data analysis. Data analysis was done by utilizing IBM SPSS (Statistical Package for Social Sciences) Statistics version 25 (IBM). Normality of the data distribution was tested using a Shapiro–Wilk test. Descriptive statistics such as frequencies (for categorical variable) and mean ± standard deviation (for continuous variable) was performed. Inferential statistics, such as, Mann–Whitney *U* test and Kruskal–Wallis tests were used to assess the differences among the study variables with the participants KAP score, while Spearman's rank‐order correlation coefficient was done to perceive the strength and direction of the relationship between the variables (e.g., knowledge with attitude and practice). Median KAP scores were calculated, and compared with the study variable by using Kruskal–Wallis and Mann–Whitney *U* tests. A *p* value of less than 0.05 was considered as a significant level while *p* < 0.01 was considered significant for correlation analysis (two tailed). Furthermore. *χ* ^2^ test along with binary logistic regression was done to identify the predictive factors related to vaccination status.

## 3 RESULTS

### 3.1 Demographic characteristics of the participants

"Table 1" depicts the distribution of the participants' sociodemographic characteristics. In the total sample (*n* = 807), the gender distribution was nearly equal, with 429 (53.2%) males. Because of the maximum availability on the online platform, most of the participants (70.4%) surveyed were between the ages of 20 and 29, with the mean age of 26.25 ± 9.94 years. The majority of participants were Muslim (89.1%), had a tertiary level of education (72.2%), were students (68.8%), and were single (66.8%) in terms of marital status. In addition, 35.9% of respondents lived in the divisional city, and 83.6% and 93.7% did not smoke or consume alcohol, respectively. Furthermore, 50.8% and 7.7% of the participants reported that they had the presence of comorbidity and their family members had HB.

**Table 1:** Demographic characteristics of the study population

Variables	Frequency	Percentage
Age (26.25 ± 9.94)		
10–19	83	10.3
20–29	568	70.4
30–39	65	8.1
40–49	50	6.2
50 and above	41	5.1
Gender		
Male	429	53.2
Female	378	46.8
Religion		
Islam	719	89.1
Hindus/others	88	10.9
Educational qualification		
No formal education	50	6.2
Primary school (up to Class 5)	20	2.5
Secondary school (Class 6–10)	72	8.9
Higher secondary (Class 11–12)	82	10.2
Tertiary education	583	72.2
Occupation		
Unemployed	39	4.8
Employed	102	12.6
Retired	9	1.1
Housewife	68	8.4
Student	555	68.8
Others	34	4.2
Residence area		
Village	211	26.1
Subdistrict town	159	19.7
District town	147	18.2
Divisional city	290	35.9
Marital status		
Single	539	66.8
In a relationship	60	7.4
Married	193	23.9
Divorced/widowed	15	1.9
Smoking status		
Yes	132	16.4
No	675	83.6
Alcohol consumption		
Yes	51	6.3
No	756	93.7
Family member suffering from hepatitis B		
Yes	62	7.7
No	745	92.3
Comorbidity		
Yes	410	50.8
No	397	49.2

### 3.2 Knowledge about HB infection

The overall mean knowledge score for HB infection was 11.506 ± 5.403 among the general people. Table 2 depicts the relationship between the median scores of knowledge with the study variables. The knowledge score was significantly associated with the participant's age (*p* ˂ 0.001), educational qualification (*p* ˂ 0.001), occupation (*p* ˂ 0.001), residence area (*p* = 0.002), marital status (*p* ˂ 0.001), and participants family member suffering from HB infection (*p* = 0.002) (Table 2). Knowledge was higher among the young people aged between 10 and 29 (median = 12, interquartile range [IQR] = 12 and median = 13, IQR = 7); who had tertiary education (median = 13, IQR = 7); who were employed (median = 13.5, IQR = 8); living in divisional city (median = 13, IQR = 7); were single (median = 13, IQR = 7); and whose family members were suffering from HB (median = 14, IQR = 4) infection.

**Table 2:** Distribution of participant's knowledge, attitude, and practice in respect to hepatitis B infection

Variables	Knowledge			Attitude			Practice		
	(11.506 ± 5.403)			(5.435 ± 1.038)			(4.252 ± 1.776)		
	Median	IQR	Rank	Median	IQR	Rank	Median	IQR	Rank
Age^a^									
10–19	13	12	398.11	5	1	383.31	4	3	357.01
20–29	13	7	426.52	6	1	396.18	5	1	420.93
30–39	11	9	377.45	6	2	500.79	4	3	406.70
40–49	9	11.5	335.12	5.5	1	400.39	4	4	367.98
≥50	6	11	230.00	6	1	405.18	3	3	304.29
*p* Value	**˂0.001**			**0.009**			**0.004**		
Gender^b^									
Male	13	8	417.86	5	1	388.17	4	2	416.17
Female	12	7	391.79	6	1	421.96	4	2	390.19
*p* Value	0.11			0.032			0.10		
Religion^b^									
Islam	12	9	400.22	6	1	397.25	4	2	400.81
Hindus/others	13	6.7	434.92	6	1	459.19	4	3	430.10
*p* Value	0.18			**0.014**			0.25		
Educational qualification^a^									
No formal education	3	3.25	132.06	5	2	339.57	2,5	3	205.47
Primary school	7	6.75	209.40	6	1	451.30	3	2.75	290.58
Secondary school	10	10	334.32	6	1	424.67	4	3	359.76
Higher secondary	13	8.25	410.66	5	1	369.31	4	2	399.80
Tertiary education	13	7	441.67	6	1	410.23	4	2	430.97
*p* Value	**˂0.001**			0.08			**˂0.001**		
Occupation^a^									
Unemployed	13	11	393.46	5	2	334.10	4	3	419.56
Employed	13.5	8	423.46	6	1	474.80	5	2.25	473.79
Retired	9	15.5	312.22	5	1.5	365.94	3	5	344.22
Housewife	6	9	237.93	6	1	426.15	3	2	264.79
Student	13	7	431.54	5	1	392.82	4	1	414.41
Others	8	10	264.65	6	1	419.97	3	1.25	301.16
*p* Value	**˂0.001**			**0.005**			**˂0.001**		
Residence area^a^									
Village	12	11	373.70	5	1	384.67	4	2	371.64
Subdistrict town	12	9	376.37	5	1	388.66	4	2	373.57
District town	12	7	398.62	6	1	405.47	4	2	441.14
Divisional city	13	7	443.92	6	1	425.72	4	3	425.41
*p* value	**0.002**			0.16			**0.004**		
Marital status^a^									
Single	13	7	432.72	6	1	397.23	4	1	414.53
In a relationship	12	7.75	417.37	5	1	373.38	4.5	1	458.65
Married	10	9	339.58	6	1	441.59	4	4	377.53
Divorced/widowed	3	9	147.53	5	2	286.10	2	2	147.47
*p* Value	**˂0.001**			**0.010**			**˂0.001**		
Smoking status^b^									
Yes	11	8	369.64	6	1	440.24	4	2.75	402.53
No	13	8	410.72	6	1	396.91	4	2	404.29
*p* Value	0.06			**0.04**			0.93		
Alcohol consumption^b^									
Yes	10.5	10	363.53	6	1	412.88	5	4	462.60
No	12.5	8	406.73	6	1	403.40	4	2	400.05
*p* Value	0.19			0.76			0.059		
Comorbidity^b^									
Yes	12	8	403.00	6	1	427.01	4.5	2	422.04
No	13	8	404.02	5	1	379.16	4	2	385.60
*p* Value	0.95			**0.002**			**0.02**		
Family member suffering from hepatitis B^b^									
Yes	14	4	492.96	6	1	419.95	5	3	529.92
No	12	9	396.60	6	1	402.67	4	2	393.52
*p* Value	**0.002**			0.55			**˂0.001**		

*Note*: Bold values are statistically significant.

Abbreviation: IQR, interquartile range, third quartile (Q3) – first quartile (Q1).

^a^ Kruskal–Wallis *H* test.

^b^ Mann–Whitney *U* test.

### 3.3 Attitude about HB infection

The overall mean score of attitudes toward HB infection was 5.435 ± 1.038 among the general people. Table 2 depicts the relationship between the median scores of attitude with the study variables. The median attitude score was statistically significant with the age (*p* = 0.009), gender (*p* = 0.032), religion (*p* = 0.014), occupation (*p* = 0.005), marital status (*p* = 0.010), smoking status (*p* = 0.04), and comorbidity (*p* = 0.002) (Table 2).

### 3.4 Practice of HB infection

The overall mean practice score for HB infection among the general people was 4.252 ± 1.776. The practice level was significantly higher among the participant aged 20–29 (median = 5, IQR = 1, *p* = 0.004); with secondary to tertiary level education (*p* ˂ 0.001), employed (median = 5, IQR = 2.25, *p* ˂ 0.001); living in village to divisional city (*p* = 0.004). Furthermore, the practice was more common among those who were in a relationship (median = 4.5, IQR = 1, *p* ˂ 0.001); who had comorbidity (median = 4.5, IQR = 2, *p* = 0.02); and whose family member had HB (median = 5, IQR = 3, *p* ˂ 0.001) (Table 2).

### 3.5 Vaccination status against HB infection

**Figure 1:**
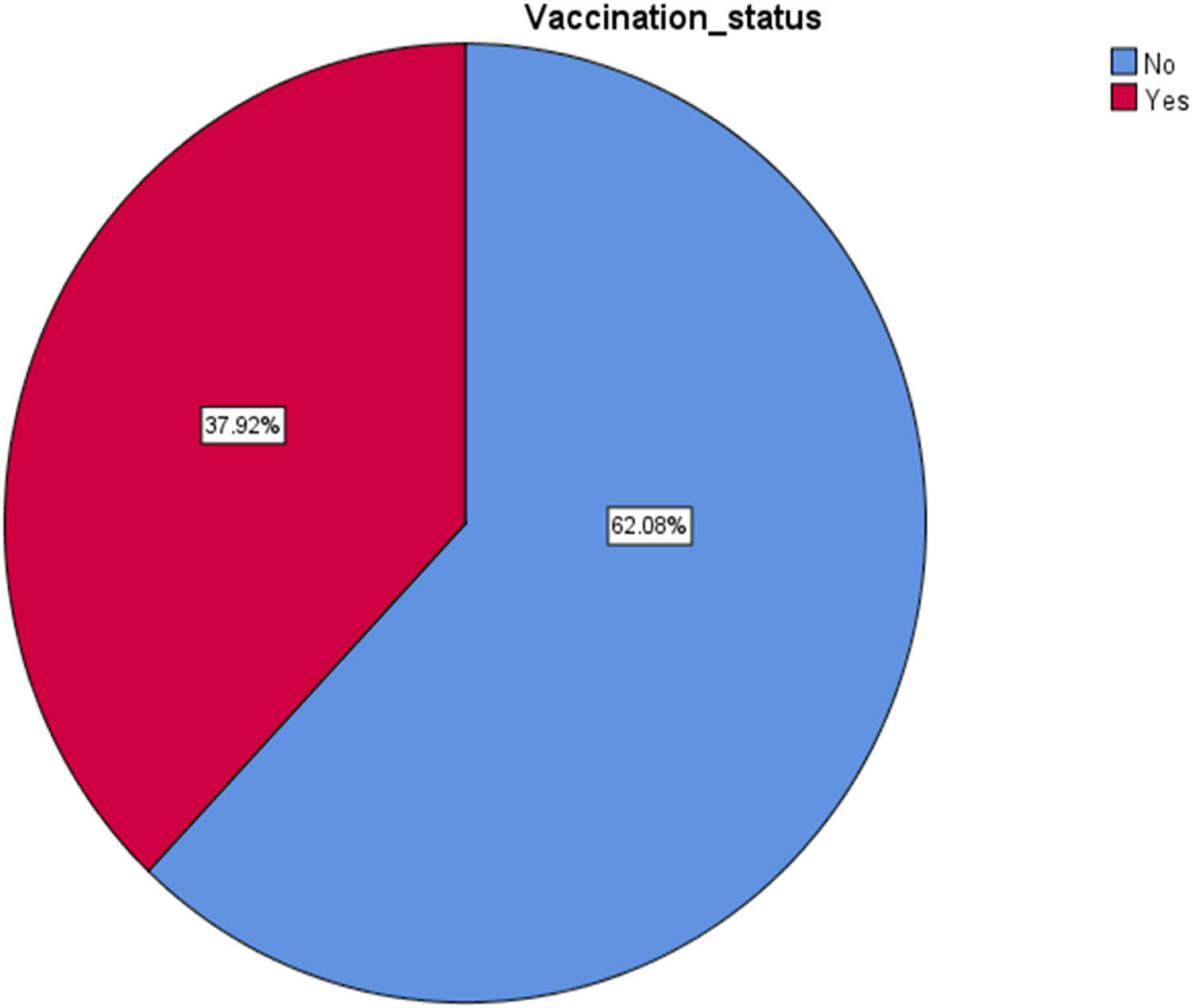
Vaccination status about hepatitis B among general people in Bangladesh

Among the 807 respondents, 37.9 percent (*n* = 306) had received a vaccine against HBV, while 62.1 percent (*n* = 501) had not (Figure 1).

The predictive factors that influenced vaccination status were depicted in (Table 3). Age was significantly associated with vaccination status where 75.6% of the participants aged between 50 and above did not receive a vaccine against HB compared to other age groups (odds ratio [OR] = 1, *p* = 0.02]. A lower rate of vaccination status was also observed among the participants who were Muslims [OR = 0.573, 95% CI = (0.368–0.894), *p* = 0.01]; who had no formal education [OR = 0.226, 95% CI = (0.100–0.511), *p* < 0.001)]; living in a rural area [OR = 0.445, 95% CI = (0.303–0.652), *p* < 0.001)] and who were divorced or widowed in terms of relationship status. Furthermore, participants who had comorbidities (OR = 1, *p* = 0.02) and had no family members suffering from HB (OR = 1, *p* = 0.04) reported lower vaccination status significantly.

**Table 3:** Association between study variables and vaccination status about hepatitis B

Predictor	Vaccination status		*χ* ^2^ test value	*p* Value	Odds ratio (95% confidence interval)
	Yes	No			
Age					
10–19	22 (26.5)	61 (73.5)	10.989	**0.02**	1.118 (0.471–2.651)
20–29	227 (40)	341 (60)			2.064 (0.992–4.292)
30–39	30 (46.2)	35 (53.8)			2.657 (1.120‐–6.302)
40–49	17 (34)	33 (66)			1.597 (0.635–4.016)
50 and above	10 (24.4)	31 (75.6)			Reference
Gender					
Female	148 (39.2)	230 (60.8)	0.461	0.49	1.104 (0.803–1.467)
Male	158 (36.8)	271 (63.2)			(Reference)
Religion					
Islam	262 (36.4)	457 (63.6)	6.125	**0.013**	0.573 (0.368–0.894)
Hindus/others	44 (50)	44 (50)			Reference
Educational qualification					
No formal education	7 (14)	43 (86)	20.010	**<0.001**	0.226 (0.100–0.511)
Primary school	6 (30)	14 (70)			0.595 (0.226–1.571)
Secondary school	26 (36.1)	46 (63.9)			0.785 (0.472–1.305)
Higher secondary	23 (28)	59 (72)			0.542 (0.326–0.901)
Tertiary education	244 (41.9)	339 (58.1)			Reference
Occupation					
Unemployed	15 (38.5)	24 (61.5)	16.768	**0.005**	2.917 (0.978–8.697)
Employed	49 (48)	53 (52)			4.314 (1.646–11.307)
Retired	3 (33.3)	6 (66.7)			2.333 (0.451–12.060)
Housewife	16 (23.5)	52 (76.5)			1.436 (0.505–4.082)
Student	217 (39.1)	338 (60.9)			2.996 (1.220–7.355)
Others	6 (17.6)	28 (82.4)			Reference
Residence area					
Village	56 (26.5)	155 (73.5)	21.584	**<0.001**	0.445 (0.303–0.652)
Subdistrict town	54 (34)	105 (66)			0.633 (0.424–0.946)
District town	66 (44.9)	81 (55.1)			1.003 (0.673–1.495)
Divisional city	130 (44.8)	160 (55.2)			Reference
Marital status					
Single	203 (37.7)	336 (62.3)	8.907	**0.03**	3.927 (0.877–17.580)
In a relationship	31 (51.7)	29 (48.3)			6.948 (1.442–33.480)
Married	70 (36.3)	123 (63.7)			3.699 (0.811–16.869)
Divorced/widowed	2 (13.3)	13 (86.7)			Reference
Smoking status					
Yes	51 (38.6)	81 (61.4)	0.035	0.85	1.037 (0.707–1.522)
No	255 (37.8)	420 (62.2)			Reference
Alcohol consumption					
Yes	24 (47.1)	282 (37.3)	1.932	0.16	1.494 (0.846–2.640)
No	27 (52.9)	474 (62.7)			Reference
Comorbidity					
No	166 (41.9)	230 (58.1)	7.139	**0.02**	1.407 (1.057–1.873)
Yes	139 (33.9)	271 (66.1)			Reference
Family member suffering from hepatitis B					
Yes	31 (50)	31 (50)	4.164	**0.04**	1.709 (1.017–2.874)
No	275 (36.9)	470 (63.1)			Reference

*Note*: Bold values are statistically significant.

### 3.6 Correlation between knowledge, attitude and practice

The correlation between KAP is depicted in Table 3. Correlations in this study were interpreted using an *r* value of 0–0.25 to indicate a weak correlation, 0.25–0.5 to indicate a fair correlation, 0.5–0.75 to indicate a moderate correlation, and >0.75 to indicate a strong correlation. Positive linear correlations were found between knowledge‐attitude (*r* = 0.205, *p* < 0.01) and knowledge‐practice (*r* = 0.390, *p* < 0.01), indicating a weak and fair correlation, respectively. There was, however, no correlation between attitude and practice (Table 4).

**Table 4:** Correlation analysis between knowledge, attitude, and practice

	Knowledge	Attitude	Practice
Knowledge	1	0.205^a^	0.390^a^
Attitude	0.205^a^	1	
Practice	0.390^a^		1

^a^ Spearman's rho correlation is significant at the 0.01 level (two‐tailed).

## 4 DISCUSSION

HB is a potentially fatal global public health threat. To avert HB infection, WHO is working to raise awareness, promote partnerships, and mobilize resources to assist countries in meeting the global hepatitis elimination targets outlined in the Sustainable Development Agenda 2030, as well as to develop evidence‐based policy and data for action to prevent infection.^2^ Given the circumstances, the current study was conducted to assess the vaccination status of HB, as well as KAP and its predictive factors among the general people in Bangladesh. The study's main strength is that it provides a better understanding of the scenario of HB infection in Bangladesh, which is important for HBV prevention.

According to the study's findings, only 37.9% of the respondents had received a vaccine against HBV infection although there is a safe and effective vaccine available that provides 98%–100% protection against HB.^2^ Furthermore, the overall mean score of practice against HB infection was 4.252 ± 1.776 (out of 8). However, poor vaccination status and HB infection prevention practice indicate a lack of commitment to combating HB infection. Furthermore, poor practices may play a vital role in low vaccination rates and increase exposure to the disease as well as their vulnerability to contracting and/or spreading the disease among potential people.^22^ The present study findings suggest that more HB‐related health education, public awareness and promotion, and appropriate practice are required to eradicate HBV in Bangladesh. In terms of vaccination status, the current study showed that older participants (aged between 50 and above) received less HB vaccine. A consistent result was found compared to a prior study where about 2.1% of people who received vaccines were aged between 40 and higher.^23^ Their decreased incidence of immunization status, however, might have a particular cause. It could be due to the the vaccine's accessibility since it was first initiated in 1986.^24^ Additionally, there was a significant relationship between religion and vaccination status, with Muslims having a lower vaccination rate than those of other religious faiths. This result contrasts with a previous study's lack of a meaningful association.^23^ The decreased rate of vaccination status indicated by participants who were divorced or widowed, who had comorbidities, and whose family members did not have HB infection and it was never investigated in any Bangladeshi study.

A previous study of general population participants found no significant association between gender and KAP.^9^ In contrast with the previous study's findings, the current study yielded different results although similar to others studies.^25^, ^26^ In addition, knowledge was significantly higher among people between 10 and 29 years while in contrast with the previous study.^9^ However, the study revealed that after the age of 20–29 years, the level of knowledge among participant's drops significantly and is mostly inferior to those aged 50 and up. In terms of educational qualification, participants with a higher level of education demonstrated adequate knowledge. It was also found that with the increment in educational status, knowledge levels significantly increased among the participants. However, a prior study depicted that secondary education was the significant predictor of good practice against HB infection, which is identical with our present findings.^18^ Students reported adequate knowledge when compared to others and at the same time, employed people reported a significantly higher positive attitude and practice which could be attributed to organizational constraints such as the need to maintain regulatory enforcement of occupational health and safety standards. In contrast, less practice was found among the retired and housewife. According to a previous study, people in cities had more knowledge and practices than people in rural areas,^9^, ^27^ which is in accordance with the current findings. Besides, participants who live in a district town or divisional city rather than a village area have adequate knowledge against HBV infection. It could be due to increased media exposure and understanding of health issues such as HB infection prevention especially through access to the Internet. As a result, they have a significant advantage over their competitors who lack access to the necessary information. People who are single demonstrated more knowledge, while practice in the current study was higher among those who were in a relationship which is in contrast with other study findings in terms of HBV infection prevention.^25^ In addition, those whose family members had HB infection reported adequate knowledge and practice rather than who's not and owing to firsthand knowledge of the infection's serious consequences, which is a new finding. In addition, the presence of comorbidity significantly influences on an individual's attitude and practice, which is another new insight in this study. However, knowledge about HB infection prevention was statistically significantly associated with a positive attitude and practice toward HB infection prevention, but only at the 1% significance level. This implies that gaining sufficient knowledge may raise health awareness, allowing for the implementation of HB infection prevention activities.^25^ Although the study's main strength is that it was conducted among the general population in Bangladesh for the first time, there were several limitations. For instance, the current study was cross‐sectional and conducted through an online platform due to the outbreak of the omicron variant of the COVID‐19 pandemic. Furthermore, due to the online platform's availability, the majority of the respondents were students, limiting the generalizability of the findings. As a result, the study is nonrepresentative. However, an offline‐based follow‐up study is recommended, which would be more robust and assist the Bangladeshi government in taking the necessary steps to eradicate HB infection. In addition, we would also encourage further collaborative studies with virologists/general practitioners/transfusion medicine specialists including clinical data analysis and multiple outcomes within the very actual HB prevalence/diagnostic/lack of treatment topic.

## 5 CONCLUSIONS

The current study's findings indicate that the general people's vaccination status and understanding of HB infection control and management are inadequate. Age, religion, educational status, occupation, residence area, marital status, alcohol consumption, comorbidity status, and at least one family member infected with HB are all predictive factors for KAP. As a result, vaccination coverage should be expanded throughout the country. Furthermore, an educational campaign focusing on the aforementioned predictive factors for HB infection should be organized. It is feasible to do so with the available manpower of public health graduates. Furthermore, to manage and control HB infection, a collaborative approach between doctors, nurses, public health specialists, and healthcare assistants should be strengthened.

## AUTHOR CONTRIBUTIONS

**Ismail Hosen**: Conceptualization; data curation; formal analysis; investigation; methodology; resources; software; validation; visualization; writing – original draft; writing – review and editing. **Mst. Sabrina Moonajilin**: Conceptualization; investigation; methodology; resources; supervision; validation; visualization; writing – review and editing. **Nur Hussain**: Formal analysis; resources; software; validation; visualization; writing – review and editing.

## CONFLICT OF INTEREST

The authors declare no conflict of interest.

## TRANSPARENCY STATEMENT

The lead author Ismail Hosen affirms that this manuscript is an honest, accurate, and transparent account of the study being reported; that no important aspects of the study have been omitted; and that any discrepancies from the study as planned (and, if relevant, registered) have been explained.

## Supporting information

Supplementary information.

## Data Availability

The data can be found from the corresponding author upon request.
