# Supplementary material for: Predictive factors of vaccination status, knowledge, attitudes, and practice towards prevention of hepatitis B infection among Bangladeshi people: A cross‐sectional study
Source: Health Sci Rep. 2022 Dec 19;6(1):e1000. doi: 10.1002/hsr2.1000 (PMC9763968; doi:10.1002/hsr2.1000)
Supplement: Supplementary file 1 — Supplementary information. [file HSR2-6-e1000-s001.pdf]

Predictive factors of vaccination status, knowledge, attitudes, and practice towards prevention of hepatitis B infection among Bangladeshi people

Socio-demographic information

Your age (Write in number, e.g. 20)

…………………………..

Gender

1. Male
2. Female

Your Religion

1. Islam
2. Hindus/others

Educational qualification

1. No formal education
2. Primary school (Up to class 5)
3. Secondary school (class 6 to 10)
4. Higher secondary (class 11 to 12)
5. Tertiary education

Occupation

1. Unemployed
2. Employed
3. Retired
4. Housewife
5. Student

Current district of residence (e.g. Dhaka)

…………………………..

Which type of administrative region are you living in?

1. Village

2. Sub-district town

3. District town

4. Divisional city

Marital status

1. Single

2. Married

3. Divorced/widowed

Do you smoke?

1. Yes

2. No

Do you consume alcohol?

1. Yes

2. No

Are you suffering from any of the following health-related issues? (You can choose more than one answer) (If you do not have any, please proceed to the next question)

1. Diabetics

2. High blood pressure

3. Asthma/ Respiratory problem

4. Heart disease

5. Kidney problem

6. Cancer

7. Any other not listed/others

Do you have any family member suffering from Hepatitis B infection?

1. Yes

2. No

Have you got yourself vaccinated against Hepatitis B?

1. Yes

2. No

**20 items of Knowledge**	YES	NO
Have you ever heard of a disease termed as Hepatitis?		
Have you ever heard of a disease termed as Hepatitis B?		
Is Hepatitis B a viral disease?		
Can Hepatitis B affect liver function?		
Can Hepatitis B cause liver Cancer?		
Can Hepatitis B affect any age group?		
The early symptoms of Hepatitis B are same like cold and flu (fever, running nose, cough)		
Jaundice is one of the common symptoms of Hepatitis B?		
Are nausea, vomiting and loss of appetite common symptom of Hepatitis B?		
Are there no symptoms of the Hepatitis B in some of the patients?		
Can Hepatitis B be transmitted by un-sterilized syringes, needles and surgical instruments?		
Can Hepatitis B be transmitted by contaminated blood and blood products?		
Can Hepatitis B be transmitted by using blades of the barber/ear and nose piercing?		
Can Hepatitis B be transmitted by unsafe sex?		
Can Hepatitis B be transmitted from mother to child?		
Can Hepatitis B be transmitted by contaminated water/food prepared by person suffering with these infections?		
Is Hepatitis B curable/treatable?		
Can Hepatitis B be self-cured by body?		
Is vaccination available for Hepatitis B?		
Is specific diet is required for the treatment of Hepatitis B?		

Knowledge was assessed by giving 1 to correct answer and 0 to the wrong answer. The scale measured knowledge from maximum 20 to minimum 0. Scores < 11 were taken as poor, ≥ 11 as adequate knowledge of Hepatitis B.

Choose the answer /7 items of attitude question
**Do you think you can get Hepatitis B?**
Yes*
NO
**What would be your reaction if you found that you have Hepatitis B?**
Fear*
Shame
Surprise
Sadness
**Who would you talk to about your illness?**
Physician
Spouse
Parents
Child
Other Relatives
Friends
No one¥
**What will you do if you think that you have symptoms of Hepatitis B?**
Go to Health facility*
Go to Hakeem
Go to Homeopath
Go to Traditional healer
**If you had symptoms of Hepatitis B, at what stage you will go to the health facility?**
Own treatment fails
After 3-4 weeks of the appearance of symptoms
Soon as I realize the symptoms are of Hepatitis B*
Will not go to physician
**How expensive do you think is the diagnosis and treatment of Hepatitis B?**
Free
Reasonable
Somewhat expensive
Expensive
Don't know¥
**What worries you most if you will be diagnosed with Hepatitis B**
Fear of death
Fear of disease spread to family
Cost of treatment
Isolation from the society¥
*** Positive attitude, ¥ Negative attitude.** **Note: Attitude was assessed by giving 1 to positive and 0 to negative attitude. The scale classified attitude as positive with score >4 and negative ≤4.**

Hepatitis B Practice Items (8)	Yes	No
Have you done screening for Hepatitis B?		
Have you got yourself vaccinated against Hepatitis B?		
Do you ask for a new syringe before use?		
Do you ask for screening of blood before transfusion?		
Do you ask your barber to change blade/Or for safe equipment's for ear and nose piercing?		
In case you are diagnosed with Hepatitis B, would you go for further investigation and treatment?		
Do you avoid meeting Hepatitis B patients?		
Have you ever participated in health education program related to Hepatitis B?		
**Note: Practice was assessed by giving 1 to positive and 0 to negative attitude. The scale classified practice as good with score >5 and poor ≤5.**		
